# Precise Molecular Design of a Pair of New Regioisomerized Fluorophores With Opposite Fluorescent Properties

**DOI:** 10.3389/fchem.2021.823519

**Published:** 2022-01-20

**Authors:** Zexin Wang, Renfu Li, Li Chen, Xin Zhai, Wei Liu, Xiang Lin, Liwei Chen, Nannan Chen, Shitao Sun, Zhenli Li, Jinle Hao, Xueyuan Chen, Bin Lin, Lijun Xie

**Affiliations:** ^1^ Fujian Provincial Key Laboratory of Screening for Novel Microbial Products, Fujian Institute of Microbiology, Fuzhou, China; ^2^ Department of Medicinal Chemistry, School of Pharmaceutical Engineering, Shenyang Pharmaceutical University, Shenyang, China; ^3^ CAS Key Laboratory of Design and Assembly of Functional Nanostructures, and Fujian Key Laboratory of Nanomaterials, Fujian Institute of Research on the Structure of Matter, Chinese Academy of Sciences, Fuzhou, China; ^4^ School of Pharmacy, Fujian Medical University, Fuzhou, China; ^5^ Hengyang Medical School, Institute of Cytology and Genetics, University of South China, Hengyang, China

**Keywords:** aggregation-induced emission, aggregation-caused quenching, reigoisomerization, multi-stimuli responsive, LDs imaging

## Abstract

Aggregation-induced emission (AIE) has attracted much attention in the past 2 decades. To develop novel AIE-active materials, ACQ-to-AIE transformation *via* regioisomerization is one of the most straightforward method. However, most of the reported ACQ-to-AIE transformations are achieved by migrating bulky units. In this work, a facile conversion was realized by migrating a small pyrrolidinyl group from *para*- to *ortho*-position on the rofecoxib scaffold. As a result, a pair of new isomers named **MOX2** and **MOX4** exhibited AIE behavior and ACQ activity, respectively. Moreover, **MOX2** also showed solvatochromic, mechanochromic, and acidochromic properties with reversible multi-stimulus behavior. Single crystal X-ray analysis of **MOX2** revealed that the molecular conformation and its packing mode were responsible for the AIE emission behavior. Further investigation indicated that **MOX2** showed high lipid droplets staining selectivity. Taken together, the current work not only provides a new design philosophy for achieving ACQ-to-AIE conversion by migrating a small pyrrolidinyl group but also presents a promising candidate **MOX2** for potential applications such as in security ink, optical recording and biological applications.

## Introduction

Aggregation Induced Emission (AIE) materials have been successfully developed in different research fields since it was discovered by Tang’s group back in 2001 (Luo et al., 2001; Hong et al., 2009; Hong et al., 2011; Mei et al., 2015; Arathi et al., 2016; Mallick et al., 2017; Lou and Yang, 2020). To date, a variety of AIE luminogens (AIEgens) have been reported (Lim et al., 2016; Choi et al., 2020; Li J. et al., 2020) for their inherent characteristics of high brightness in solid states by suppressing extensive π-π stackings in their packing mode (Yuan et al., 2014; Mei et al., 2015), which would otherwise result in aggregation-caused quenching (ACQ) of the fluorophores (Hong et al., 2009; Yuan et al., 2010). These AIEgens varied greatly in terms of their chemical structures, including tetraphenylethylene (TPE) (Feng H. T. et al., 2018; La et al., 2018; Wu et al., 2018), triphenylamine (TPA) (Zhang et al., 2013; Cao et al., 2014; Zhang Y. et al., 2014), cyano-substituted diarylethene (Shen et al., 2013; Zhang X. et al., 2014; Lu et al., 2015), 2,3,4,5-tetraphenylsiloles (Chen et al., 2014), silole (Chang et al., 2015), distyrylanthrancene (Niu et al., 2015; Xiong et al., 2015), and anthracene derivatives (Lu et al., 2010; Zhang et al., 2011; Li A. et al., 2019), conjugated polymers (Ravindran and Somanathan, 2017; Wang et al., 2018), and so on. Modification of the existing AIE scaffolds is the most popular strategy for constructing new AIEgens (Mei et al., 2015). In addition to this strategy, transforming ACQ molecules into AIE-active materials also provides a direct way to design highly bright AIEgens due to the rich source of ACQ (Yuan et al., 2010; Zong et al., 2016; Liu et al., 2017; Lu et al., 2019; Li Y. et al., 2020; Pratihar et al., 2020; Wang et al., 2020; Huang et al., 2021; Long et al., 2021; Zhang et al., 2021).

Traditionally, ACQ-to-AIE transformation can be achieved by integrating bulky substituents into ACQ molecules to avoid strong π-π stackings or by introducing twisted AIEgens (Dhokale et al., 2015; Gomez-Duran et al., 2015; Feng X. et al., 2018) and propeller-shaped molecules into ACQ fluorophores (Ma et al., 2016; Wang et al., 2020). However, integration of twisted AIEgens prolongs the π system thus it would probably change the already satisfactory fluorescent properties of the original ACQ molecules. Therefore, it will be highly desirable to achieve ACQ-to-AIE conversion without extending its conjugated system too much. Some progresses have been made along this line for the past decade (Sasaki et al., 2017; Li Y. et al., 2020). Among them, ACQ-to-AIE conversion was realized by Tang’s group recently through a regioisomerization strategy via shifting a molecular rotor in the end position of a planar core of dithieno [2,3-a:3′,2′-c] benzo [i] phenazine (TBP) to the bay position (Li Y. et al., 2020). The same strategy was utilized by Xu’s group to achieve ACQ-to-AIE conversion through changing the substituted position of a benzanthrone moiety from the meta-position to the *ortho*-position on the perylenetetracarboxylic diimide core (Long et al., 2021). Similarly, Prasad’s group also realized ACQ-to-AIE transformation by changing a cyano group from the *para*-position to the meta-position on the same phenyl group (Pratihar et al., 2020). Despite the great examples above, the successful cases of the regioisomerization of small units are still rare (Pratihar et al., 2020). Therefore, it is still necessary to develop more examples of ACQ-to-AIE conversion to guide the design of novel AIEgen materials.

**FIGURE 1:**
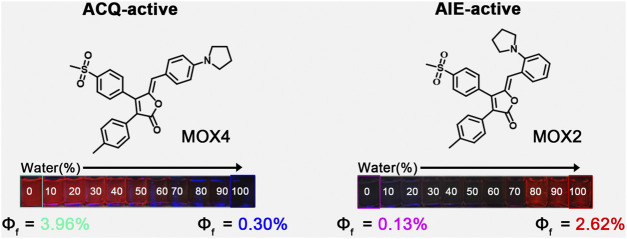
A pair of new regioisomerized fluorophores with opposite fluorescent properties.

Herein, we report our recent work on the realization of ACQ-to-AIE transformation by migrating a small pyrrolidine group from *para*-to *ortho*-position based on the rofecoxib scaffold. The resulting compounds **MOX4** with *para*-substitution showed ACQ effect while compound **MOX2** wih *ortho*-substition exhibited AIE acitivity (Figure 1). Moreover, **MOX2** also showed multi-stimulus fluorescent responsive properties, such as mechanochromic, acidochromic properties. In addition, its potential applications have been presented. This work is expected to provide a new guidance on designing AIE-type materials.

## Experimental

### Materials and Instrumentation

All commercial reagents and solvents were used as received. Compounds *p*-tolylacetic acid and 2-bromo-4′-methanesulfonyl acetophenone were purchased from Shanghai Macklin Biochemical Co., Ltd. and Shanghai Bidepharm Co., Ltd., respectively. Various benzaldehydes with different substituted groups were purchased from Shanghai Shaoyuan Co., Ltd. Reactions were magnetically stirred and monitored on a TLC Silica gel 60G F254 plate from Millipore Sigma (United States). All other reagents and solvents were purchased from Sigma-Aldrich (United States) and used without further purification, unless otherwise stated. Photoluminescence (PL) spectra were recorded on a Varioskan LUX 3020-80110. Differential scanning calorimetry (DSC) were obtained on a DSC STAR system at a heating rate of 15°C/min from 40°C to 500°C under a high purity nitrogen atmosphere. Powder X-ray diffraction (PXRD) patterns were recorded on a Rigaku (D/MaX-3B) diffractometer. Single-crystal X-ray diffraction was conducted with Bruker D8 Quesr/Venture diffraction with *λ* = 0.77Å (MoKα). Lipid droplets (LDs) imaging was performed with a Nikon Ti-E&C2 scanning unit. ^1^H NMR and ^13^C NMR spectra were measured on a Bruker AV 600 spectrometer in appropriated deuterated chloroform solution at room temperature with the solvent residual proton signal as a standard. High resolution mass spectra (HRMS) were recorded on a GCT premier CAB048 mass spectrometer operating in MALDI-TOF mode.

### Synthesis and Characterization

**SCHEME 1:**
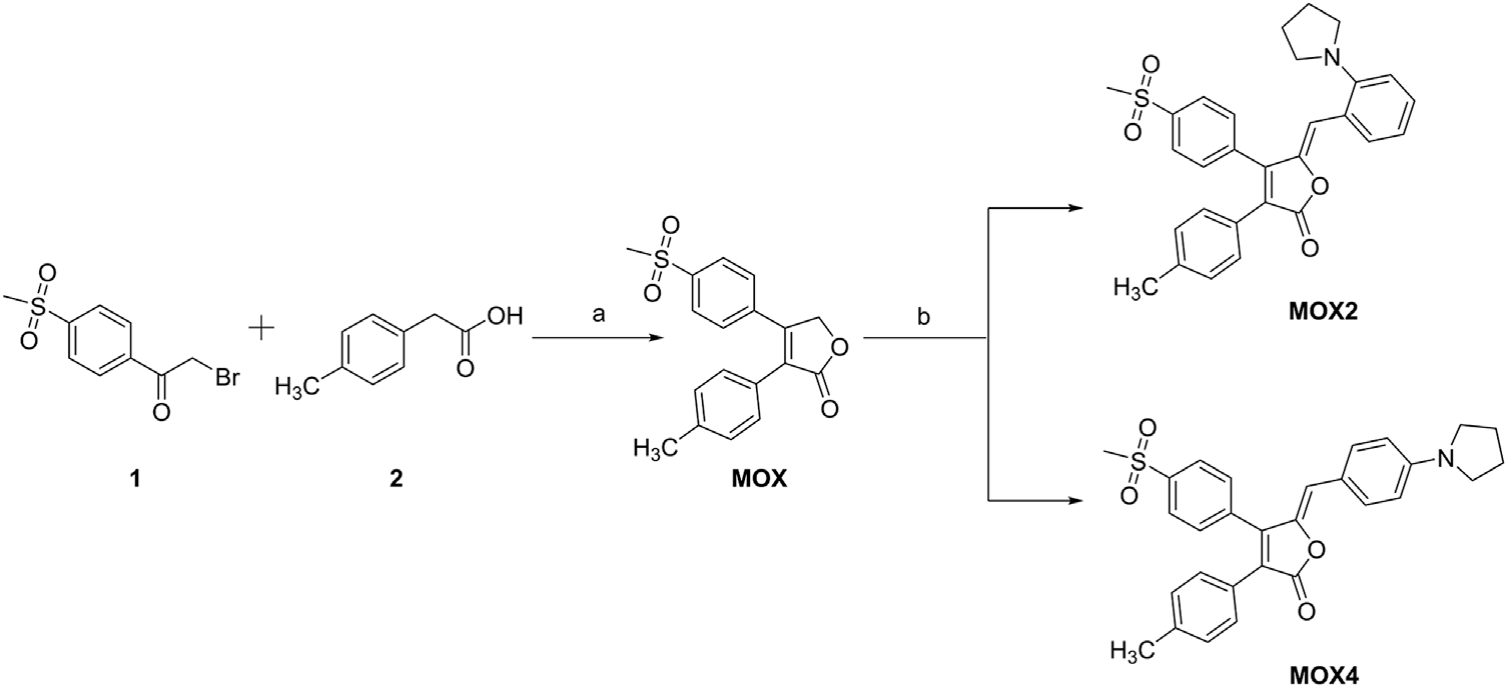
Synthetic route of **MOX2** and **MOX4**. Reaction conditions: **(A)** Et_3_N, 25°C, 1 h, DBU −5°C, 1 h; **(B)** piperidine and 2-(1-pyrrolidinyl)benzaldehyde or 4-(1-Pyrrolidinyl)benzaldehyde.

The synthetic route of compounds is outlined in Scheme 1. The key intermediate **MOX** was synthesized in a one-step reaction involving cyclization of esters *via* 2-bromo-4′-methanesulfonylacetophenone and *p*-methyl phenylacetic acid in the presence of triethylamine and DBU. Finally, the target compounds **MOX2** and **MOX4** were prepared via Knoevenagel condensation reaction. **(*General procedure*)**. Piperidine, 3 drops, was added to a mixture of 0.140 g (0.0004 mol) of intermediate (**MOX**) and 0.140 g of 2-(1-Pyrrolidinyl) bezaldehyde in 10 ml of methanol, and the mixture was stirred at room temperature for 12 h in dark atmosphere. The mixture was then cooled, and the precipitate was filtered off and washed with methanol on a filter. The yellow powder (**MOX2**, 0.1001 g) was obtained with the yield 72%. The DSC thermogram of powder **MOX2** showed only one endothermic peak that corresponds to the melting point (Tm = 203.6°C) (Supplementary Figure S12). ^1^H NMR (600 MHz, DMSO-*d*_*6*_) δ 8.09 (d, *J* = 8.3 Hz, 2H), 7.89 (d, *J* = 8.8 Hz, 1H), 7.72 (d, *J* = 8.3 Hz, 2H), 7.26 (d, *J* = 23.8 Hz, 3H), 7.17 (d, *J* = 8.1 Hz, 2H), 7.05–6.89 (m, 2H), 6.04 (s, 4H), 3.06 (s, 3H), 2.28 (s, 1H), 1.76 (s, 4H). ^13^C NMR (151 MHz, DMSO-*d*_*6*_) δ 168.3, 150.3, 148.8, 146.6, 142.1, 139.2, 136.1, 131.5, 130.6, 130.5, 129.5, 129.3, 128.0, 126.5, 124.8, 123.2, 120.6, 116.8, 111.9, 52.6, 43.7, 24.9, 21.4.HR-MS (ESI): calced for C_29_H_27_NO_4_S: 486.1734 [(M + H)^+^]), found:486.1813. The red powder (**MOX4**, 0.1230 g) was synthesized following the general procedure with the yield 88%. The DSC thermogram of powder **MOX4** showed only one endothermic peak that corresponds to the melting point (Tm = 231.3°C) (Supplementary Figure S12). ^1^H NMR (600 MHz, DMSO-*d*_*6*_) δ 8.05 (d, *J* = 8.1 Hz, 2H), 7.67 (dd, *J* = 8.2, 3.9 Hz, 4H), 7.20 (d, *J* = 8.0 Hz, 2H), 7.13 (d, *J* = 8.0 Hz, 2H), 6.61 (d, *J* = 8.7 Hz, 1H), 5.94 (s, 1H), 3.33 (s, 3H), 2.27 (s, 4H), 1.97 (s, 4H). ^13^C NMR (151 MHz, DMSO-*d*_*6*_) δ 168.4, 148.7, 148.6, 144.2, 141.9, 138.5, 136.3, 133.0, 130.8, 129.5, 129.2, 128.0, 127.0, 122.4, 120.5, 115.2, 112.5, 47.7, 43.8, 25.4, 21.3. HR-MS (ESI): calced for C_29_H_27_NO_4_S:486.1734 [(M + H)^+^], found:486.1771. The final products were characterized by ^1^H NMR, ^13^C NMR, high-resolution mass spectrometry and single-crystal X-ray diffraction. The relevant data are collected from the original spectra and listed in the Support Information (Supplementary Figures S1–S6).

## Results and Discussion

### Absorption and PL Intensity in Solution

**FIGURE 2:**
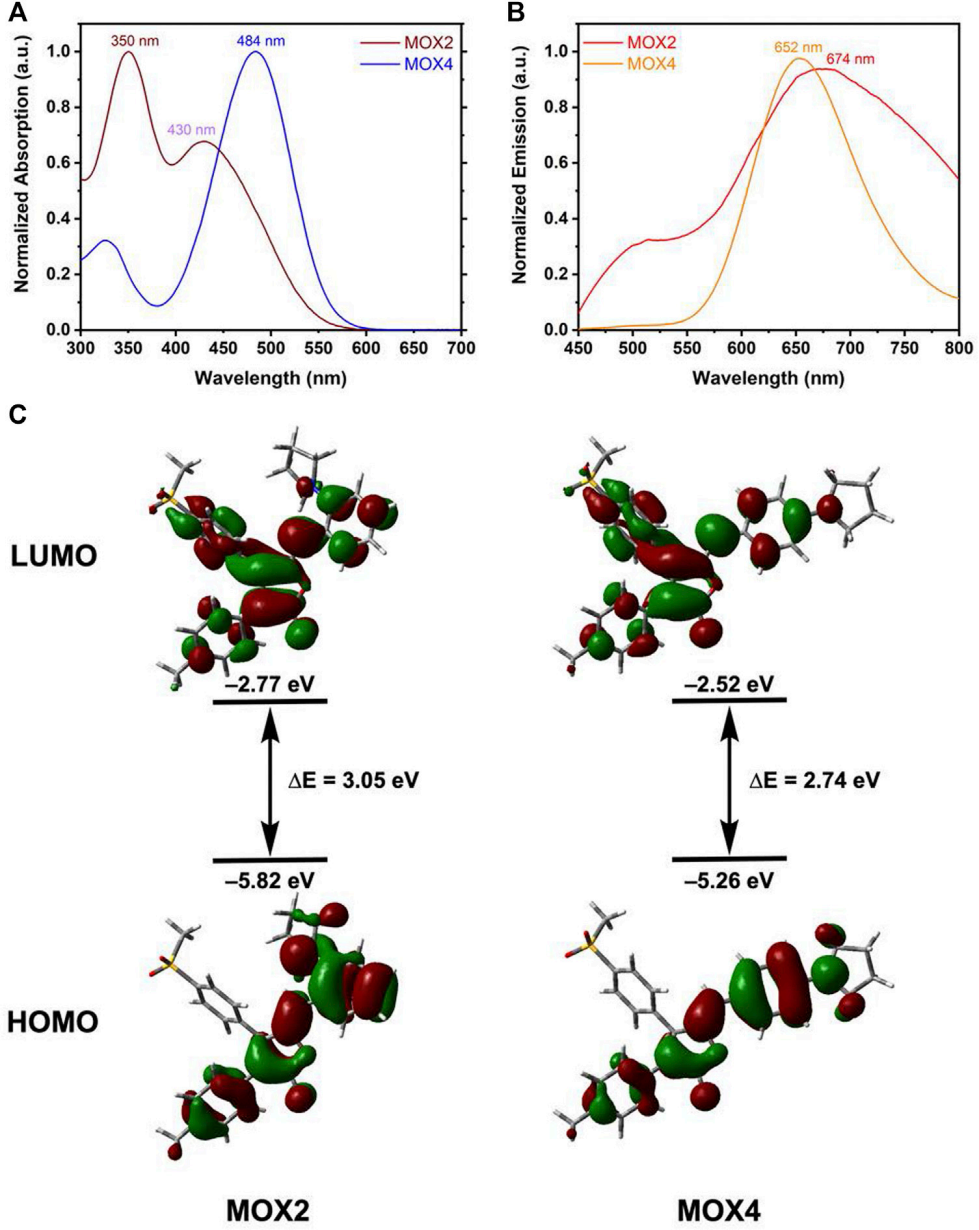
Normalized absorption **(A)** and PL **(B)** spectra of **MOX2** and **MOX4** dyes in DMSO (50 μM). **(C)** The optimized structure of **MOX4**, **MOX2**, and their HOMO and LUMO at the B3LYP/6-31++G (2 d, p) level.

As displayed in Figure 2A, **MOX2** had two absorption bands at 350 and 430 nm, respectively. The high energy band was attributed to the π-π transition while the low energy band was probably caused by the internal charge transfer (ICT) (Pati et al., 2013; Pati et al., 2015). In contrast, **MOX4** has only one maximum absorption band at 484 nm. In terms of emission, **MOX2** had a maximum PL intensity at 674 nm, slightly red-shifted compared to 652 nm of **MOX4** (Figure 2B). Another attractive feature of **MOX2** was its large Stokes shifts (13,675 cm^−1^) in dimethyl sulfoxide (DMSO) as displayed in Supplementary Table S1, which may be beneficial for overcoming the self-quenching of traditional fluorophores (Li P. et al., 2019). We carried out an experiment of 50 μM concentration, this intensity was consistent with the Beer-Lambert’s plot (Supplementary Figure S7). To provide insight into regioisomerization of the pyrrolidine group in compounds **MOX2** and **MOX4** on their photophysical properties, density functional theory (DFT) calculations at the B3LYP/6-311++G (2 d,p) level were carried out (Lee et al., 1988). The ICT was evident in comparison between the highest occupied molecular orbital (HOMO) and the lowest unoccupied molecular orbital (LUMO). The energy band gap was determined to be 3.05 and 2.75 eV for **MOX2** and **MOX4**, respectively, which was consistent with our experimental absorption data (Figure 2C).

### Solvatochromic Properties

**FIGURE 3:**
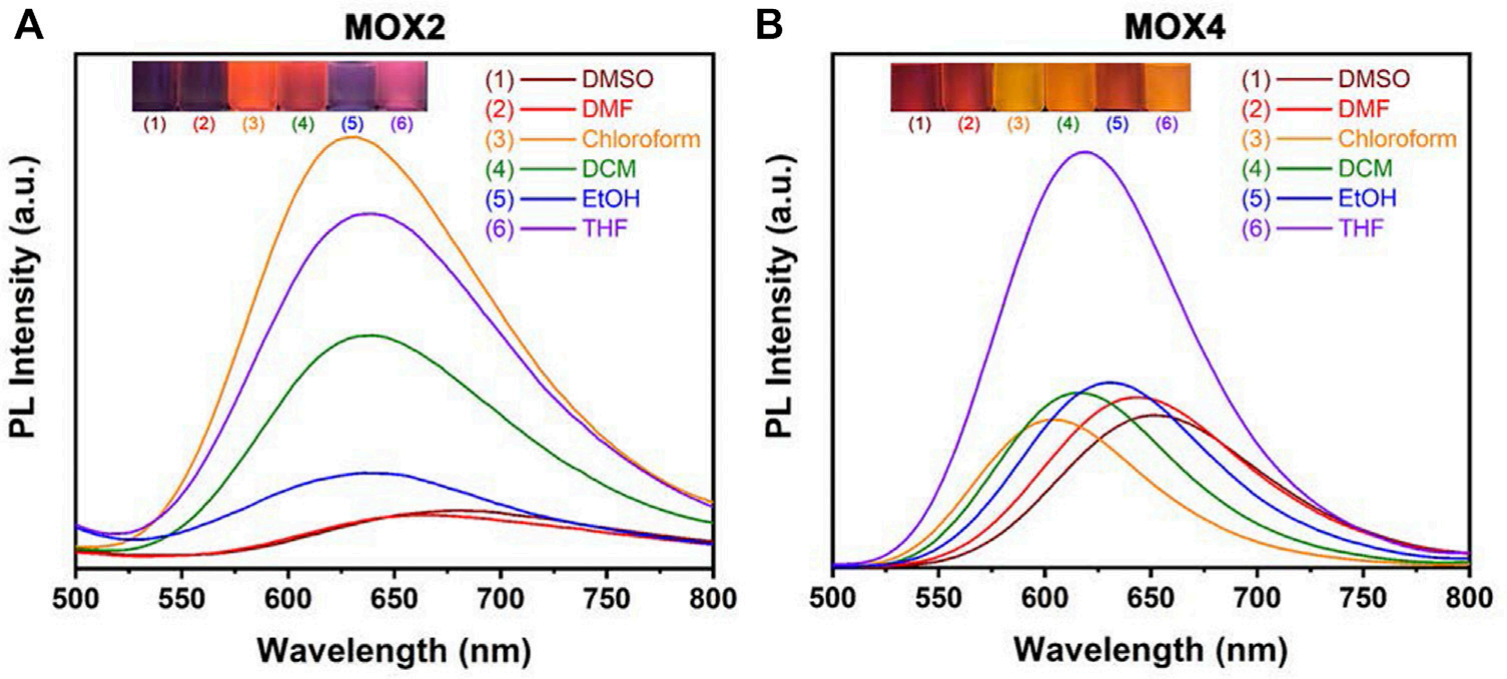
PL spectra of compounds **MOX2 (A)** and **MOX4 (B)** in various solvents (50 µM), such as DMSO, DMF, chloroform, DCM, EtOH, THF, respectively. Inset: photograph of **MOX2 (A)** and **MOX4 (B)** in various solvents with UV irradiation (*λ* = 365 nm).

The UV-vis absorption and PL spectra of **MOX2** and **MOX4** were measured in different solvents, such as DMSO, dimethylformamide (DMF), chloroform, dichloromethane (DCM), ethanol (EtOH), tetrahydrofuran (THF). The photophysical data were summarized in Supplementary Table S1. As the solvent varied from chloroform to DMSO, the *λ*_*abs*_ of **MOX2**, and **MOX4** displayed minor changes, indicating that their ground states are hardly affected by the solvent polarity (Supplementary Figure S8). In contrast, the PL spectra of **MOX2** and **MOX4** exhibited different maximum wavelengths in different solvents (Figure 3). With the solvent polarity changed from chloroform to DMSO, the *λ*_*em*_ of **MOX2**, and **MOX4** are red-shifted of 40 and 58 nm, respectively. Furthermore, the PL intensity of **MOX2** was gradually weakened with increased polarity of the solvent, indicating that the PL intensity of **MOX2** was highly dependent on the polarity of solvents.

### ACQ and AIE Properties of MOX2 and MOX4

**FIGURE 4:**
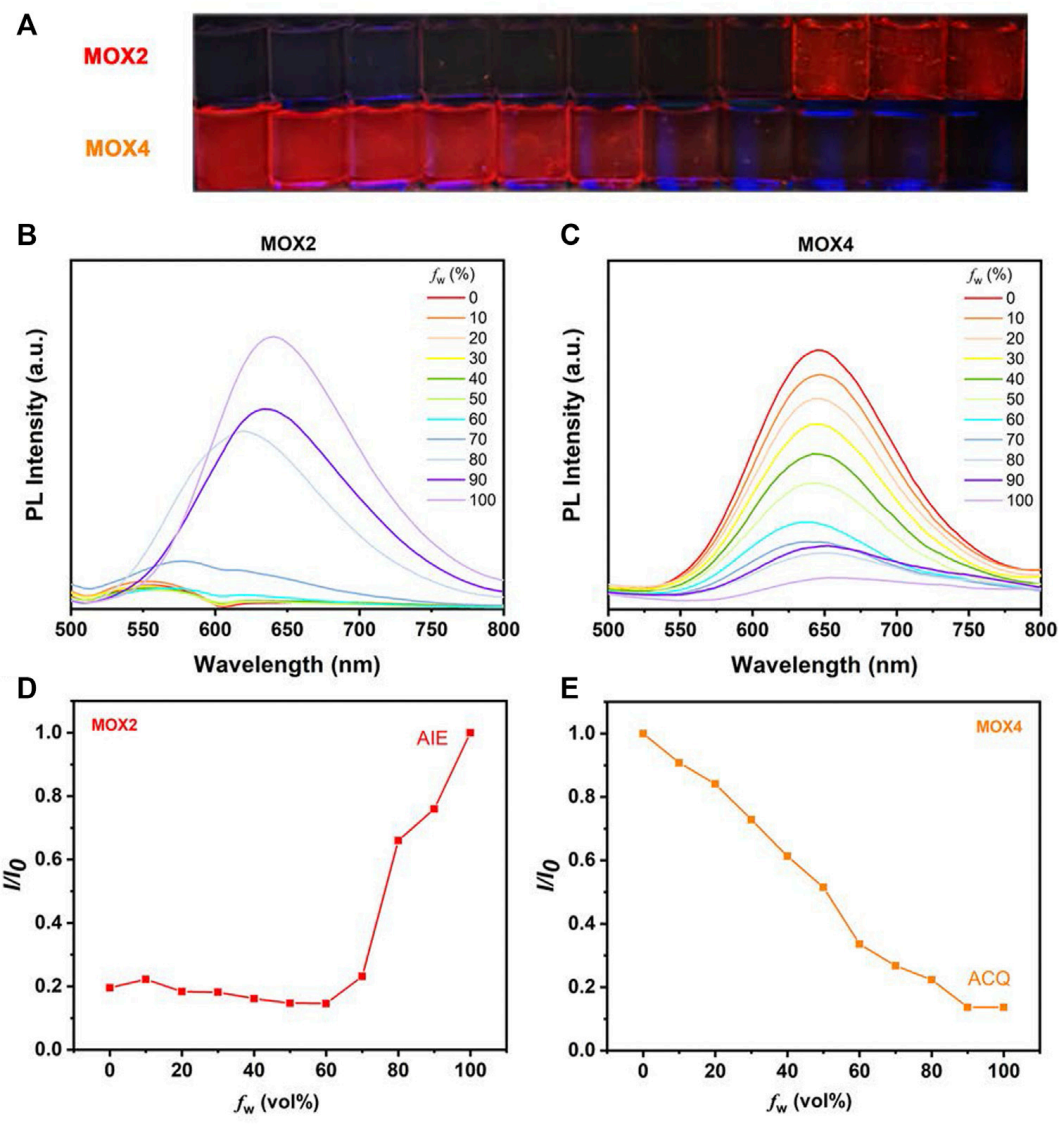
**(A)** The photos in different solvent states of **MOX2** and **MOX4** at 365 nm (10 µM). **(B)** The **MOX2** PL spectra as a function of the water fraction in DMSO. **(C)** The **MOX4** PL spectra as a function of the water fraction in DMSO. **(D)** Variation in PL intensity (*I/I*_*0*_) of **MOX2** with *f*_w_ (%), where I_0_ is the luminescence intensity in pure H_2_O. **(E)** Variation in PL intensity (*I/I*_*0*_) of **MOX4** with *f*_w_ (%), where I_0_ is the luminescence intensity in pure DMSO.

To demonstrate the feasibility of the present strategy, the photophysical properties of the two isomers were performed to monitor the PL intensity fluctuation of the molecules in DMSO/water mixtures with various water volume fractions (*f*_w_) as shown in Figure 4. The absorption spectra as a function of the water fraction of **MOX2** and **MOX4** in DMSO as displayed in Supplementary Figure S9. As shown in Figure 4C, molecule **MOX4** displayed strong emission in DMSO due to the presence of a large π-conjugated planar structure. As *f*_*w*_ was increased from 0 to 100 vol%, the PL intensity was decreased gradually, and the quantum yield (QY) of **MOX4** decreased significantly from DMSO (3.96%) to water (0.30%) (Table 1). This result demonstrated that compound **MOX4** has typical ACQ effect. On the contrary, **MOX2** exhibited week emission in DMSO (Figure 4B). In the water fraction range (*f*_w_ = 0–60 vol%), the PL intensity was still weak. However, when *f*_w_ reached 80 vol%, the PL intensity was increased significantly, which was ascribed to the aggregation of the compound (Figure 4D) (Liu et al., 2016; Sun et al., 2018; Gao et al., 2019). Moreover, **MOX2** gave a low QY in pure DMSO (0.13%) but a high QY in pure water (2.62%) (Table 1). Therefore, **MOX2** was AIE-active. The distinct difference in solid-state QY suggested a significant effect of the pyrrolidinyl position as regioisomers on the fluorescent properties.

**TABLE 1:** Photoluminescence QY of **MOX4** and **MOX2**.

Compounds	QY (%)
**MOX4** (0% water)	3.96
**MOX4** (100% water)	0.30
**MOX2** (0% water)	0.13
**MOX2** (100% water)	2.62

### Crystal Analysis of MOX2

**FIGURE 5:**
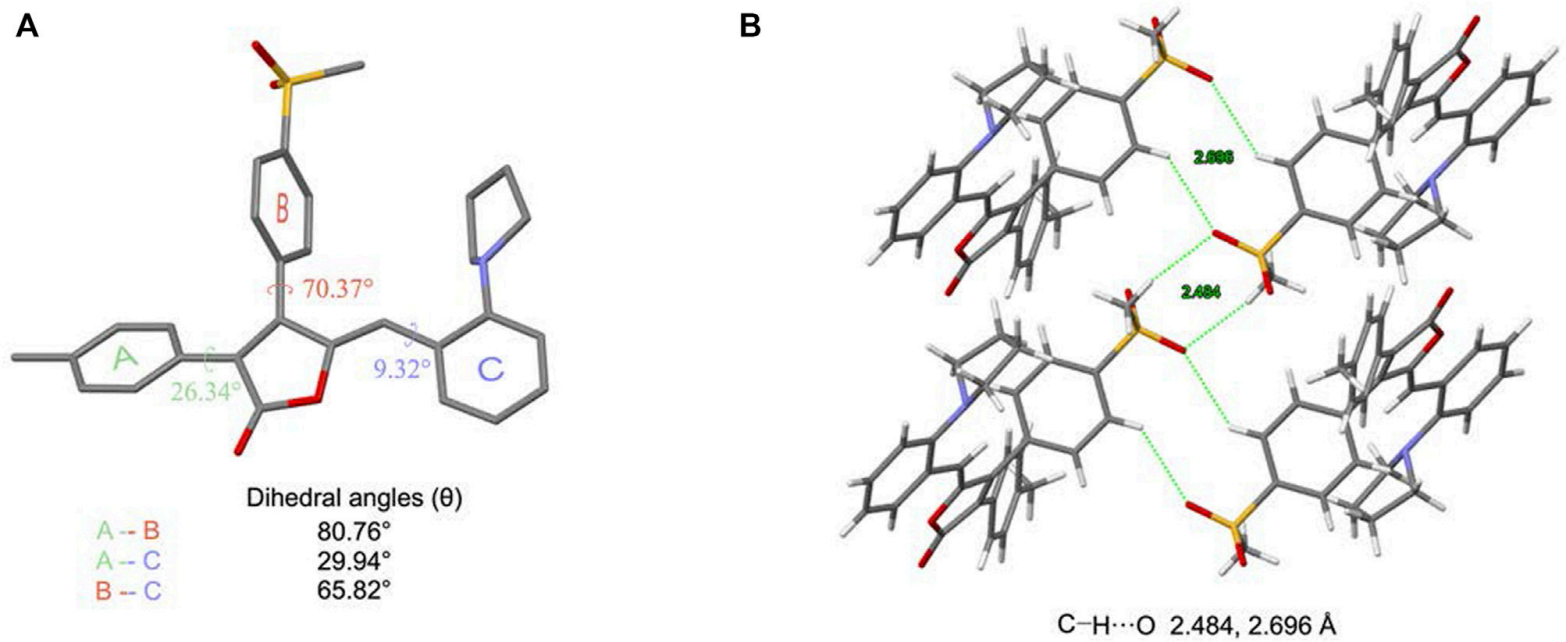
Crystal structure of **MOX2**. **(A)** a single molecule in the crystal strucure and relevant dihedral angles; **(B)** a tetramer in the crystal structure.

To better understand the origin of the AIE property, the single crystal of **MOX2** was obtained (CCDC 2107729) and its molecular packing was analyzed as follows. The crystal data and structure refinement for **MOX2** as displayed in Supplementary Table S2. As shown in Figure 5A, the dihedral angles between A-B, A-C, and B-C planes are 80.76°, 29.94°, and 65.82°, respectively. In addition, **MOX2** adopted a loose packing mode with weaker intermolecular C–H∙∙∙O (2.484 Å, 2.696 Å, 2.633 Å, 2.659 Å) interactions as shown in Figure 5B; Supplementary Figure S10. The existence of these intermolecular interactions helped rigidify the molecular conformation, thus restricting the intramolecular rotation of the phenyl group. Moreover, the non-radiative relaxation process could be largely prohibited and significant emission enhancement was observed in its solid form, yielding the remarkable AIE phenomenon. It is worth noting that such relatively weak intermolecular interactions coculd be easily destroyed upon exposure to external mechanical force, giving rise to the mechanochromic properties as studied below. The single crystal data of **MOX2** was displayed in the Supporting Information (Supplementary Table S2).

### Mechanochromic Properties of MOX2

**FIGURE 6:**
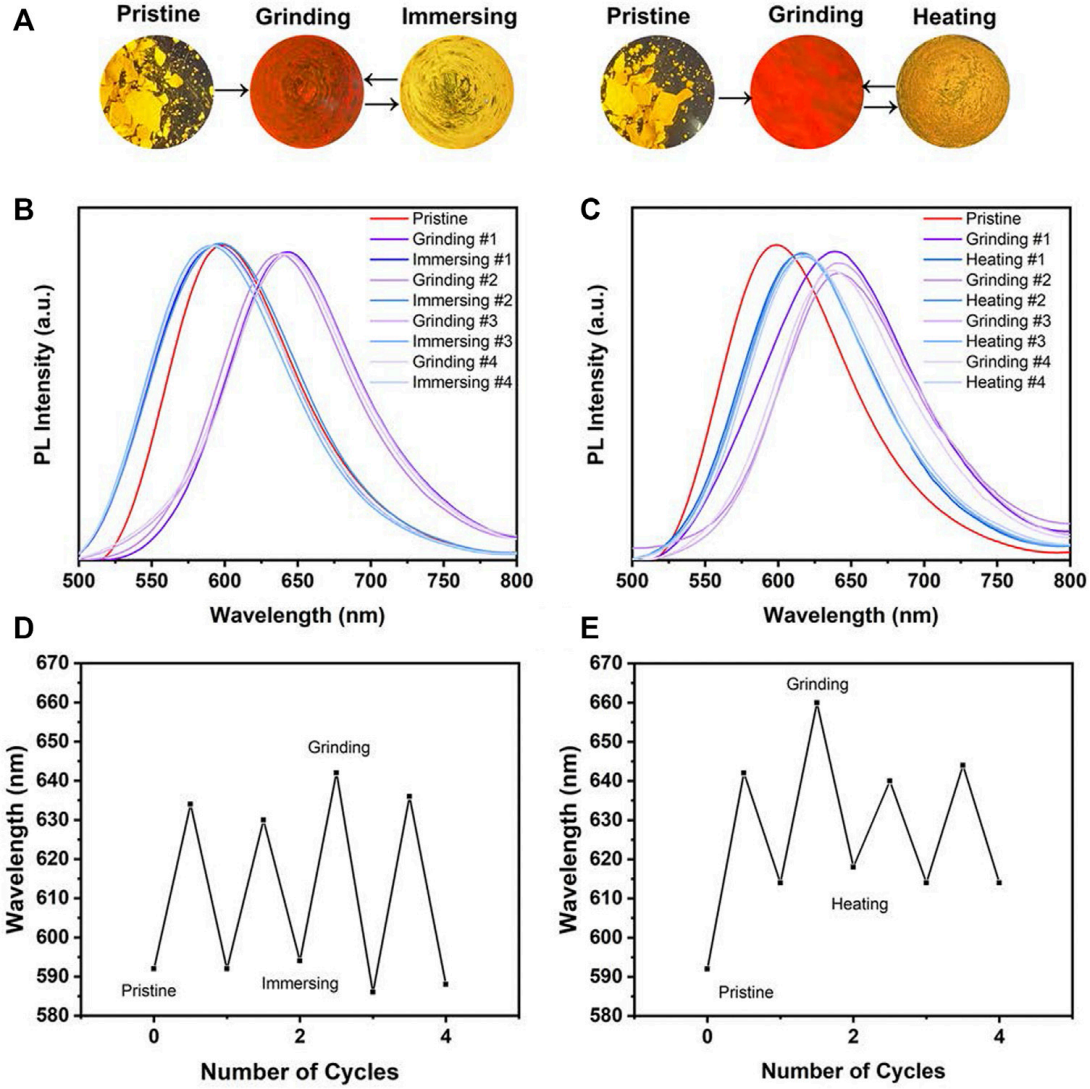
**(A)** PL spectra of **MOX2** powder under alternating grinding, immersing and heating; Images were taken under a 365 nm hand-held UV lamp. Change in the PL spectrum of **MOX2** in grinding-immersing **(B)** and grinding-heating **(D)** circles. Repeated switching of the emission wavelength in grinding-immersing **(C)** and grinding-heating **(E)** circles.

**FIGURE 7:**
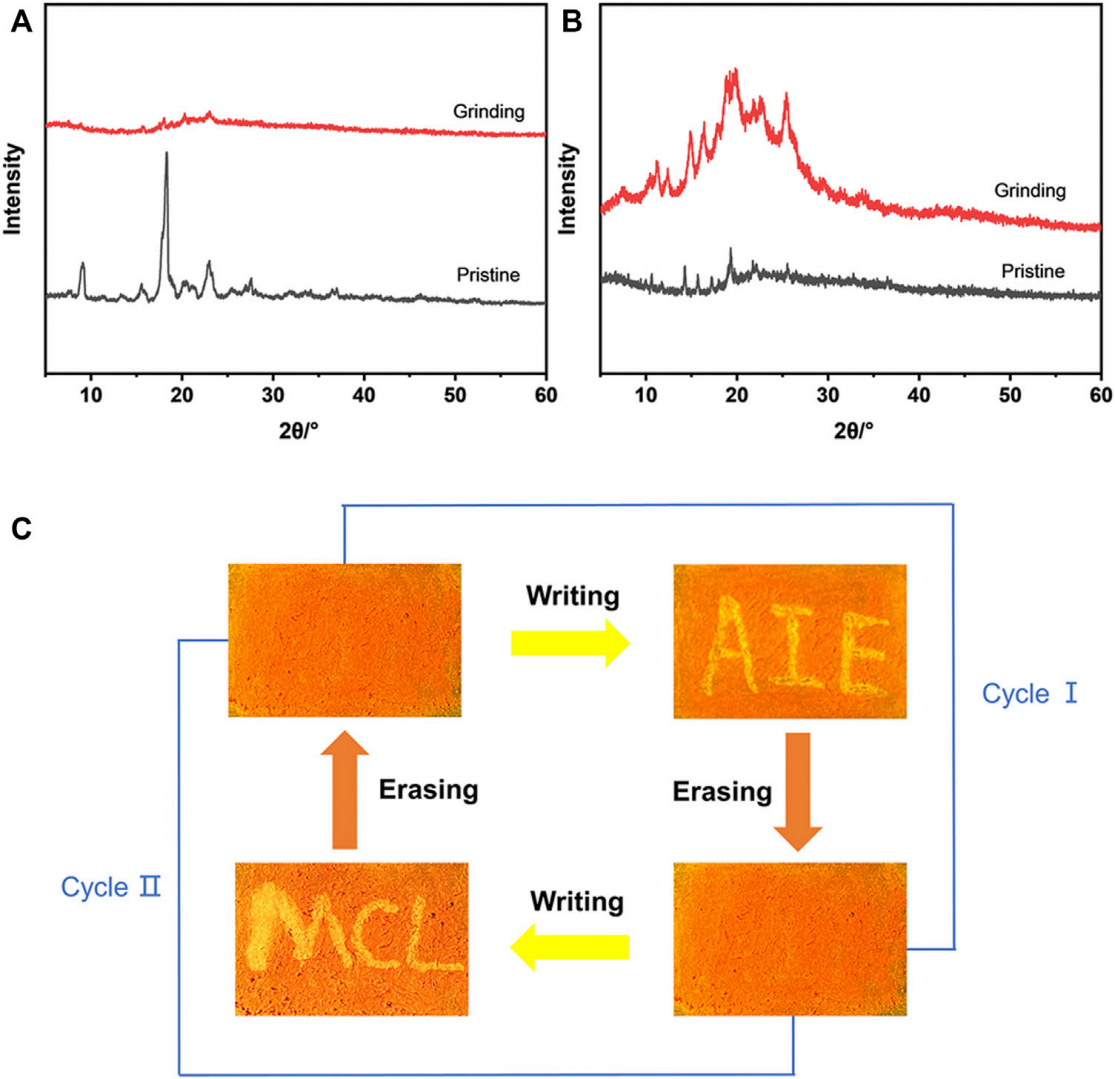
**(A)** PXRD patterns of compound **MOX2** in different states: pristine (black line), grinding (red line). **(B)** PXRD patterns of compound **MOX4** in different states: pristine (black line), grinding (red line). **(C)** A reversible data recording device of **MOX2** based on its mechanochromic phosphorescence.

Due to the AIE characteristics of **MOX2**, its mechanochromic luminescence (MCL) behaviors was investigated. As shown in Figure 6, the pristine **MOX2** powder emitted strong yellow fluorescence with a maximum at 592 nm. The emission peak red-shifted to 660 nm after grinding with red emission under 365 nm light. The spectral shift value was 68 nm (Table 2). The emission of the ground sample was restored to its original yellow color by immersing of acetone for 2 s or heated at 50°C within 10 min (Figure 6A). Moreover, fluorescence color conversion could be repeated numerous times (Figures 6B–E) without fatigue (Chan et al., 2014). To gain insight into the MCL phenomenon, PXRD and DSC experiments were conducted on the pristine, grinding, immersing, and heating solids of **MOX2** and **MOX4** (Figure 7). It showed that the intensity of the diffraction peaks observed for powdered crystalline **MOX2** significantly decreased upon grinding, which indicated the loss of crystallinity. The diffraction peaks of **MOX4** still existed after heavily grinding. Furthermore, **MOX2** exhibited greater redshift than **MOX4** after grinding (Supplementary Figure S11). The transition from a crystalline structure to an amorphous state upon grinding was further confirmed by DSC experiments. In a DSC measurement of grinding **MOX2** and **MOX4** (Supplementary Figure S12), exothermic peaks that corresponded to the cold-crystallization transition **MOX2** (T = 107.7°C) and **MOX4** (T = 155.1°C) were observed followed by endothermic peaks that corresponded to the melting point of powder **MOX2** (T = 203.6°C) and **MOX4** (T = 231.3°C), respectively. These experimental results suggested that the transformation between crystalline structure and the amorphous state was responsible for the observed MCL behavior upon external stimuli.

**TABLE 2:** Peak emission wavelengths (nm) of solid-state **MOX2** under different conditions.

Compound	*λ*_*pristine*_ (nm)	*λ*_*grinding*_ (nm)	*λ*_*immersing*_ (nm)	*λ*_*heating*_ (nm)	△*λ* (nm)
**MOX2**	592	660	586	614	68

△λ = λpristine– λas-prepared.

By exploiting the excellent MCL behavior and good reversibility of **MOX2**, a simple rewritable phosphorescence data recording device was fabricated (Figure 7C) (Gundu et al., 2017). The specific reversible procedure is as follows. First, the original sample was put into a mortar and ground as the grinding sample. Next, it was spread on a filter paper to make a thin film, showing orange emission under UV excitation. Then, the characters “AIE” were written on the thin film with a writing brush with the acetone solvent as the “ink”. Due to the change of the emission color, the yellow-emitting letters could be clearly seen. Next, the “AIE” was erased by using a cotton swab to make it completely disappear, resulting in the recovery of the original orange thin film. New letters “MCL” could be written again with the “writing brush” of the acetone solvent, which could still be removed by using a cotton swab according to the above method. This writing-erasing process can be repeated for several cycles. Based on this, **MOX2** could be a potential fluorescent material for applications as repeated writing.

### Sensing Properties Toward Protonic Acids

**FIGURE 8:**
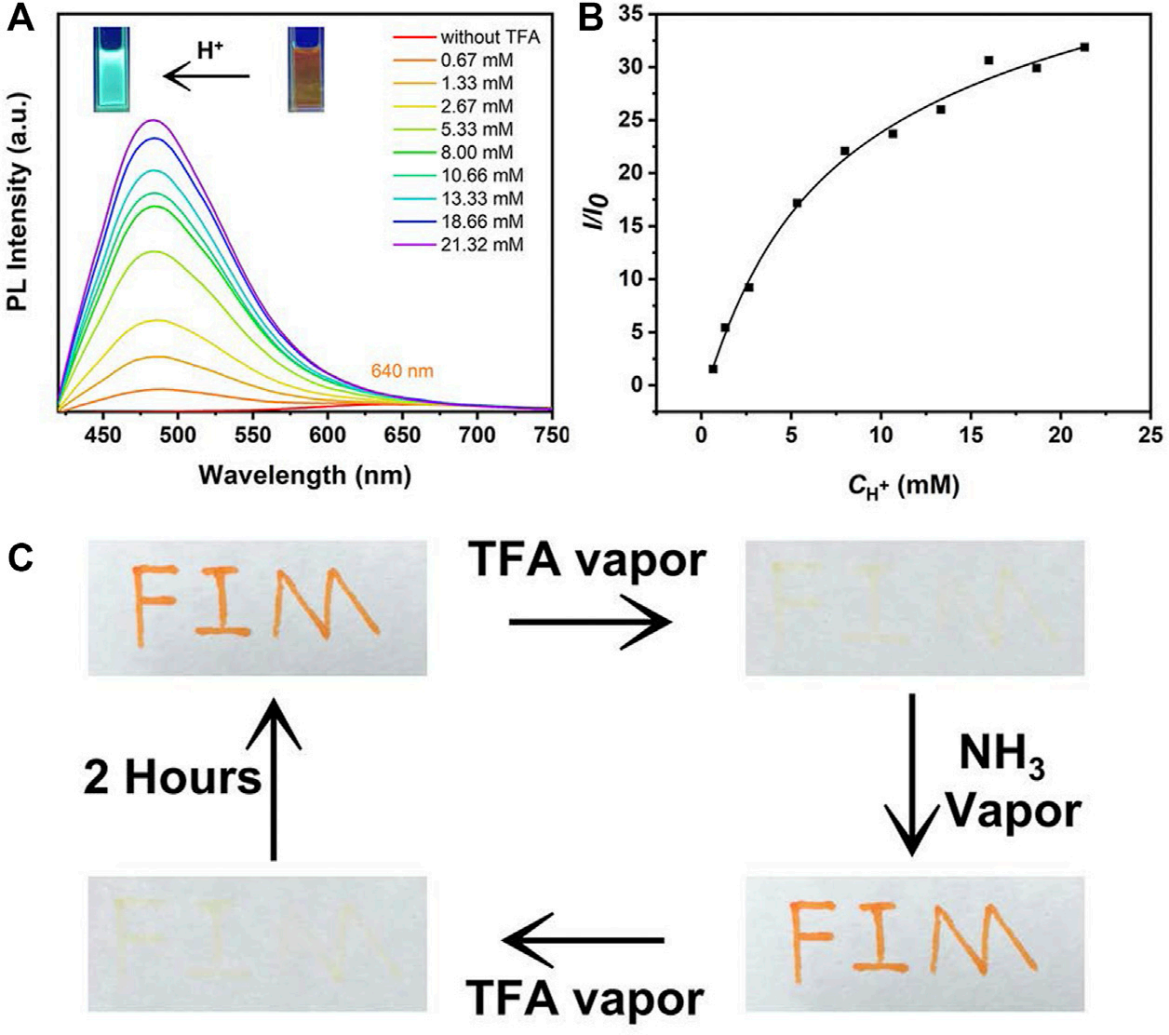
**(A)** PL spectra of **MOX2** (100 μM) in methanol solvent with different concentration (0.67-21.32 mM) of TFA. Photographs pf **MOX2** (100 μM) in methanol solvent before and after acidification at 365 nm UV light showed in the inset. **(B)** The plot and linear fitting of PL intensity at 484 nm versus TFA (0.67-21.32 mM). **(C)** Photographs of filter papers coated with **MOX2** (10 mM) in DCM solvent under different conditions.

Next, the sensing properties of **MOX2** was exploited because the pyrrolidine unit can be protonated. The solution of **MOX2** was prepared in DCM at a concentration of 1 mM. This solution was added dropwise to a filter paper. At this point, the filter paper showed a dark red color under 365 nm illumination. Then after fumigation with trifluoroacetic acid (TFA) for 1 min, at the very beginning, the filter paper exhibited light blue under 365 nm illumination. With the passage of time, the light blue faded, and then the orange began to appear (Supplementary Figure S13). Until 480 min, the blue was completely gone. The color of the filter paper sheet returned to orange (Supplementary Figure S13) (Chen et al., 2018). Meanwhile, as shown in Figure 8A, to investigate the possible acidochromic properties of **MOX2**, the spectral response ability of **MOX2** towards TFA in methanol solution (1.0 × 10^−4^ M) was also measured. When TFA was gradually added to the methanol solution of **MOX2**, the yellow solution by degrees converted into a light blue one (Figure 8A inset). Upon addition of TFA, the color of **MOX2** in methanol solvent changed from orange to blue under 365 nm illumination, and a new blue-shifted peak formed in the PL spectrum (Figure 8A) (Cao et al., 2019; Cao et al., 2021). With the concentration of TFA increased from 0 to 21.32 mM, pronounced changes in emission suggested that **MOX2** had been protonated by TFA. The ratio of PL intensity had a quadratic relationship with TFA concentration in a certain range of TFA concentrations (Figure 8B) (Cao et al., 2020). As displayed in Figure 8C, we further explored the acidochromic behavior of **MOX2** for making a rewritable media by coating the solution on a filter paper. The letters were written on filter paper were stable, which could be erased only on fuming with TFA. Fumigation with ammonia could quickly return the sample to the initial color. Fumigation with TFA again can be quickly wiped off. The color would gradually return to its initial appearance after 2 h. This demonstrated that **MOX2** has the potential to be used as security link.

### Lipid Droplets Imaging in Living Cells

**FIGURE 9:**
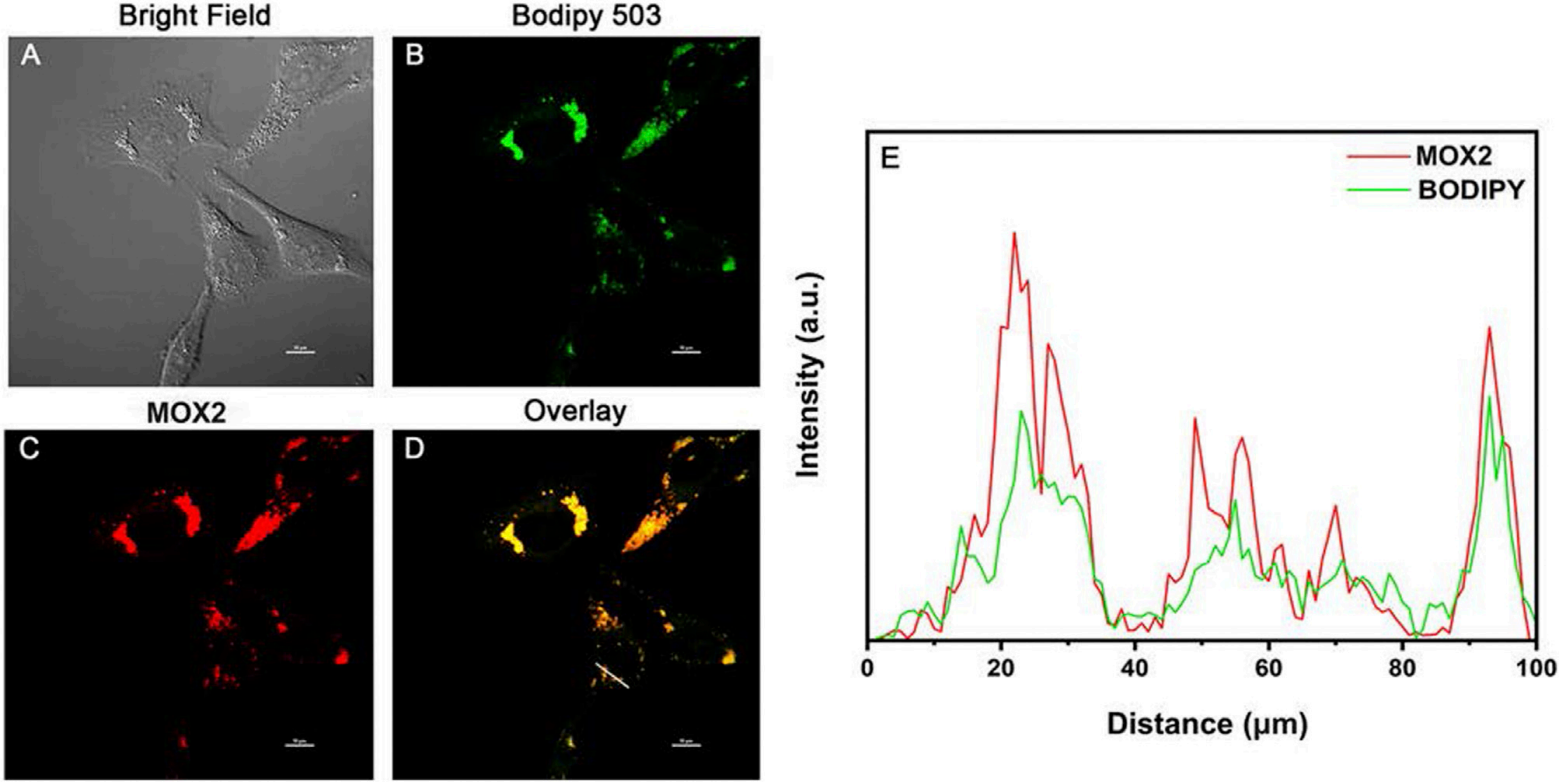
Colocalization confocal imaging of living HeLa cells stained with **MOX2** and BODIPY 503: **(A)** bright field image; **(B)** imaging channel of BODIPY 503 (*λ*_*ex*_ = 488 nm; *λ*_*em*_ = 500–540 nm); **(C)** imaging channel of **MOX2** (*λ*_*ex*_ = 488 nm; *λ*_*em*_ = 600–640 nm); **(D)** overlay image. Scale bar: 10 µm. **(E)** Red and green lines represent the fluorescence of **MOX2** and BODIPY, respectively.

Lipid droplets (LDs) are key organelles which are closely related to living status and death process of living cells (Kuerschner et al., 2008; Walther and Farese, 2009). Before cell imaging experiments, the potential cytotoxicity of **MOX2** and **MOX4** was evaluated using the MTT assay and low cytotoxicity of them was observed (Supplementary Figure S14). We first conducted co-staining experiments of LDs in living HeLa cells (Figure 9). The cells were stained with BODIPY 503 (1 × 10^−5^ M) and **MOX2** (1 × 10^–5^ M) for 0.5 h, and were imaged in green channel (*λ*_*ex*_ = 488 nm, *λ*_*em*_ = 500–540 nm) and red channel (*λ*_*ex*_ = 488 nm; *λ*_*em*_ = 600–640 nm), respectively. The two channels are well overlapped with each other. This result indicated the good LDs selectivity of **MOX2**. When HeLa cells were incubated with **MOX2** for 30 min at 37°C, strong red fluorescence signals were observed from inside cells shown in Supplementary Figure S15. This was not surprising for the far-red emitting compound **MOX2**, which showed a bright fluorescence image *in vitro*.

## Conclusion

In this work, ACQ-to-AIE transformation was achieved by migrating a small pyrrolidinyl group from *para*- to *ortho*-position in a new molecular system, in which the *para*-isomer **MOX4** showed the ACQ behavior and the *ortho*-isomer **MOX2** is AIE-active. Moreover, compound **MOX2** exhibited mechanochromic properties and pressure sensing ability. PXRD and DSC revealed that the MCL mechanism was ascribed to the transformation among crystalline state and the amorphous state by grinding. Furthermore, investigation of the acidochromic behavior of **MOX2** revealed that it could be further used as security link. In further biological experimental study, **MOX2** could be utilized as a fluorescent probe for LDs imaging in living cells. This work provides a novel strategy to achieve the ACQ-to-AIE transformation, which will provide a new guideline for future design of new AIEgens from ACQ molecules.

## Data Availability

The original contributions presented in the study are included in the article/Supplementary Material, further inquiries can be directed to the corresponding authors.
