# Supplementary material for: Precise Molecular Design of a Pair of New Regioisomerized Fluorophores With Opposite Fluorescent Properties
Source: Front Chem. 2022 Jan 20;9:823519. doi: 10.3389/fchem.2021.823519 (PMC8811449; doi:10.3389/fchem.2021.823519)
Supplement: Supplementary file 1 [file DataSheet1.docx]

Supplementary Material

Table of Contents

| Section | Title | Page |
| --- | --- | --- |
| Figure S1-S6 | Characterization of compounds | 2 |
| Figure S7 | Beer-Lambert’s plot and linear fitting of **MOX2** and **MOX4** | 5 |
| Figure S8 | The normalized absorption spectra of compounds in different solvents. | 5 |
| Table S1 | The photo-physical data of **MOX2** and **MOX4** in different solvents | 5 |
| Figure S9 | Aggregation-induced emission | 6 |
| Figure S10 | Molecular conformation of **MOX2** | 7 |
| Table S2 | Crystal data and structure refinement for **MOX2** | 7 |
| Figure S11 | Normalized solid-state PL spectra of **MOX2**, **MOX4** in the pristine state, grinding state | 9 |
| Figure S12 | DSC curves of **MOX2** and **MOX4** | 9 |
| Figure S13 | Sensing properties toward protonic acids | 9 |
| Figure S14 | Cell Viability assay | 10 |
| Figure S15 | Lipid droplets (LDs) of **MOX2** | 10 |

**Characterization of MOX2**

**
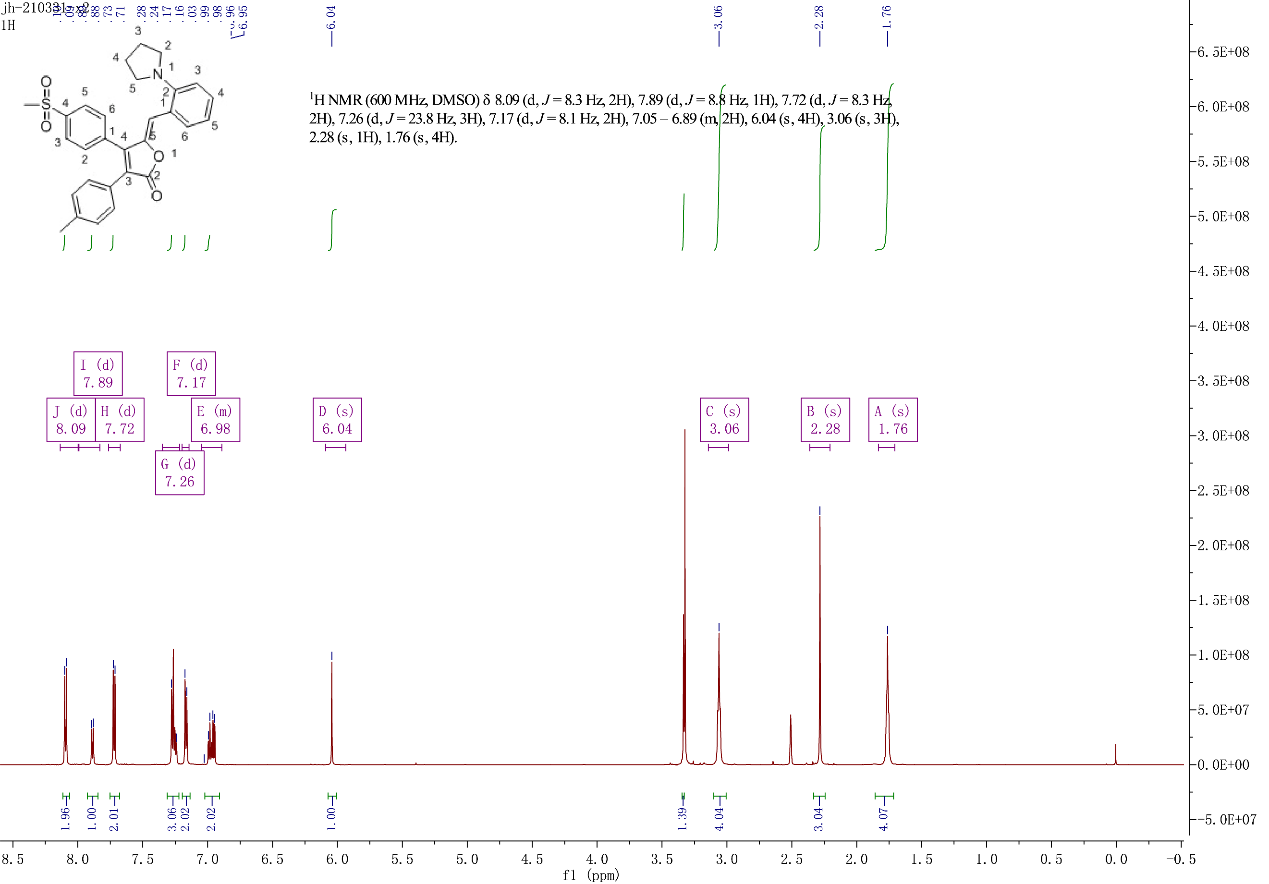
**

**Figure S1.** ^1^H NMR spectrum of **MOX2** in DMSO-*d_6_*.


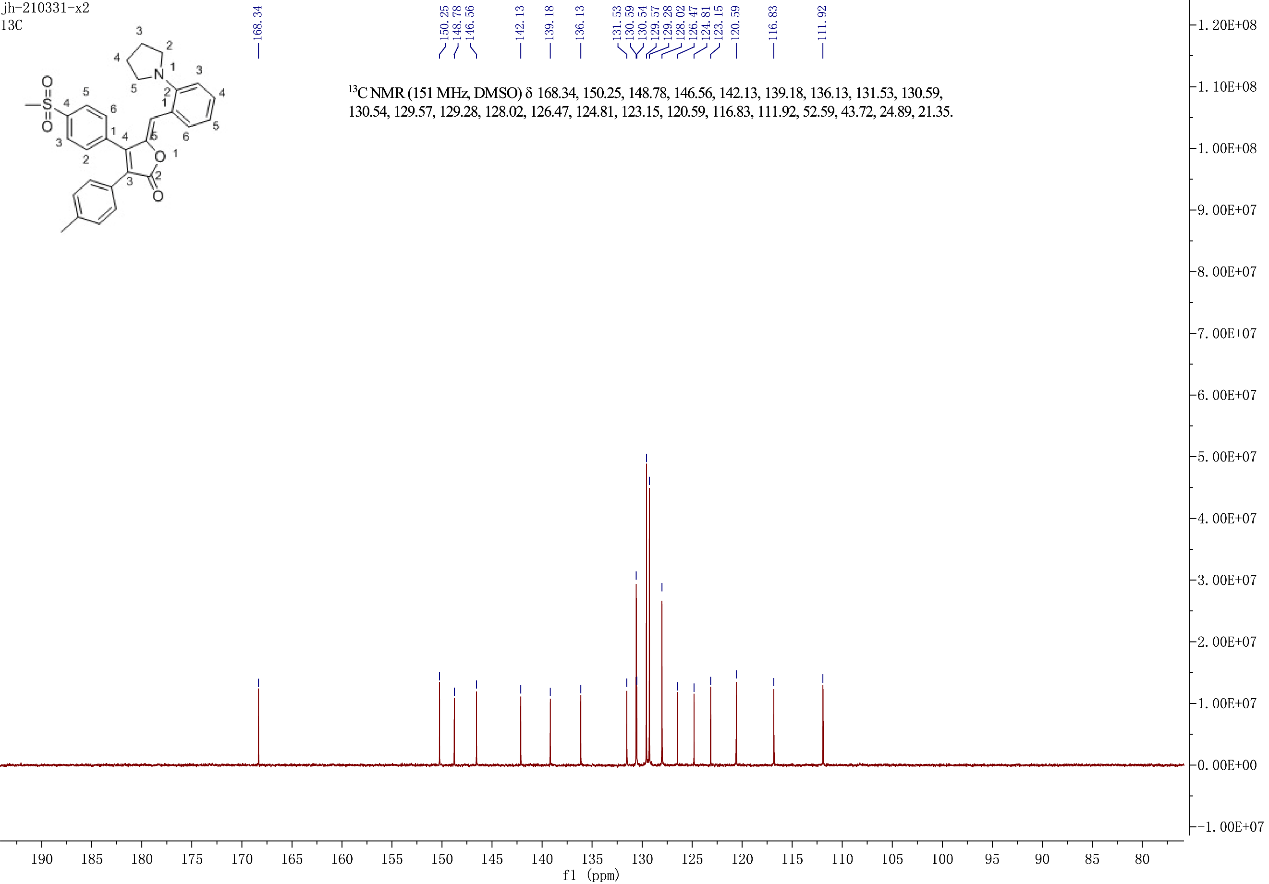


**Figure S2.** ^13^C NMR spectrum of **MOX2** in DMSO-*d_6_*.


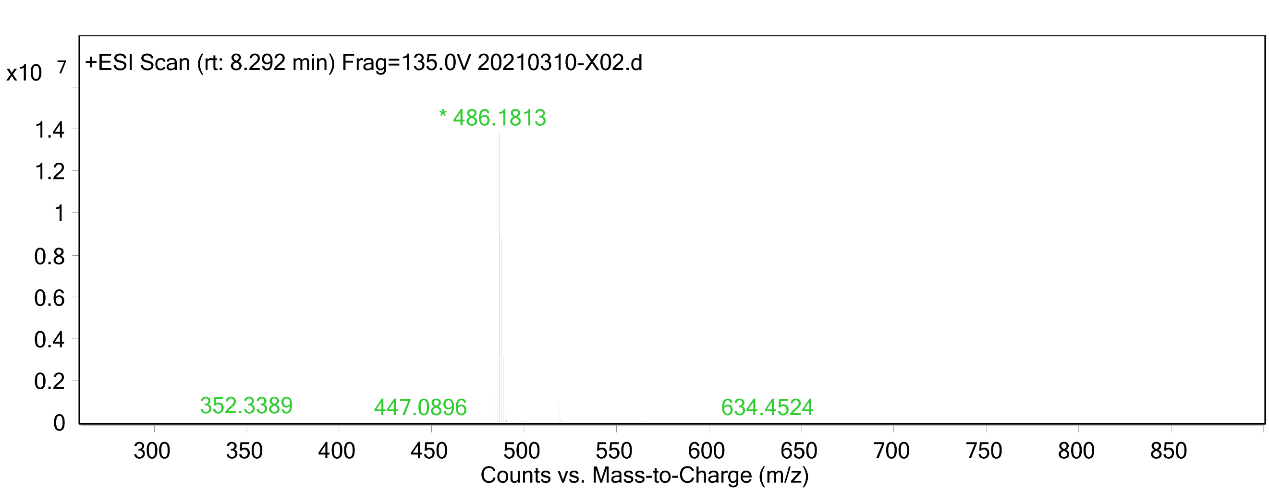


**Figure S3.** HRMS spectrum of **MOX2**

**Characterization of MOX4**


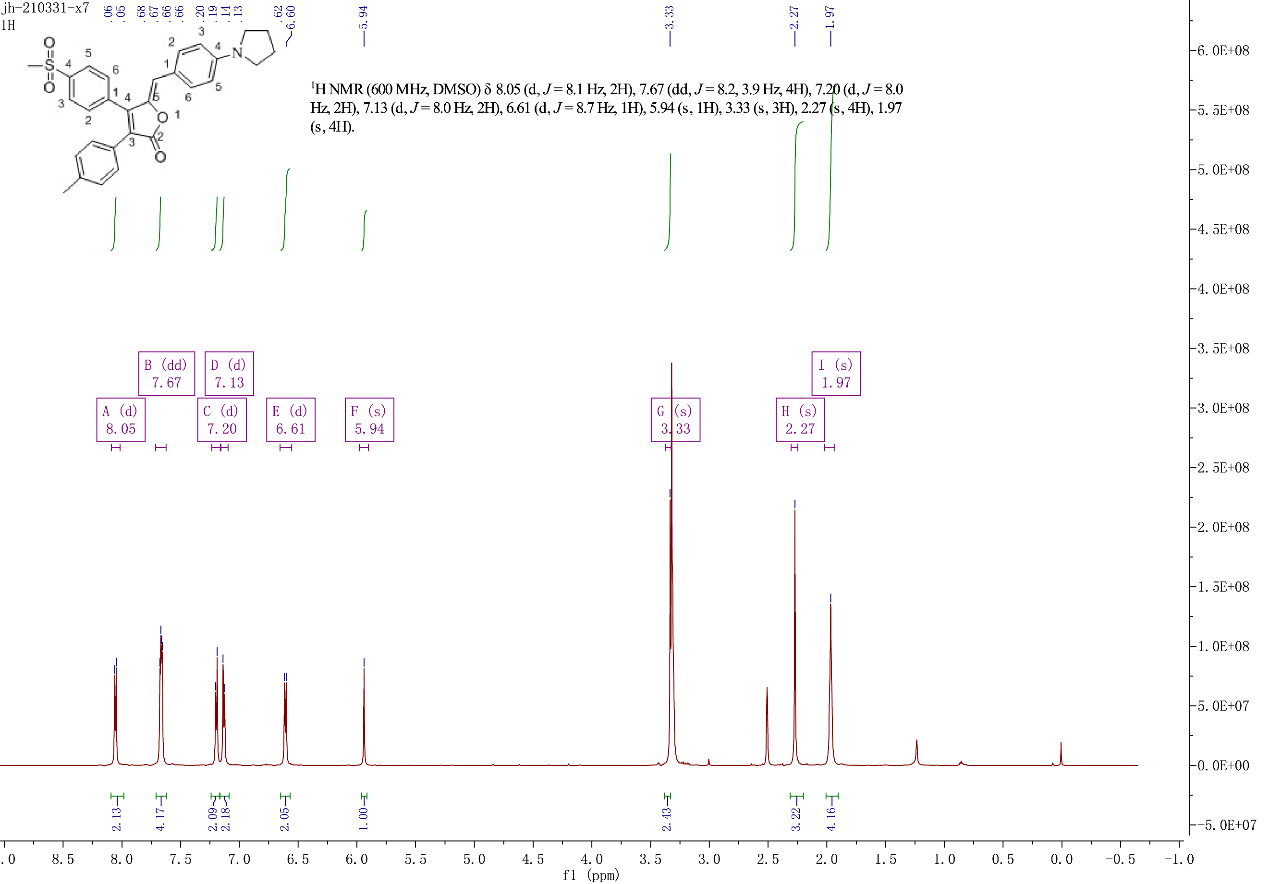


**Figure S4.** ^1^H NMR spectrum of **MOX4** in DMSO-*d_6_*.


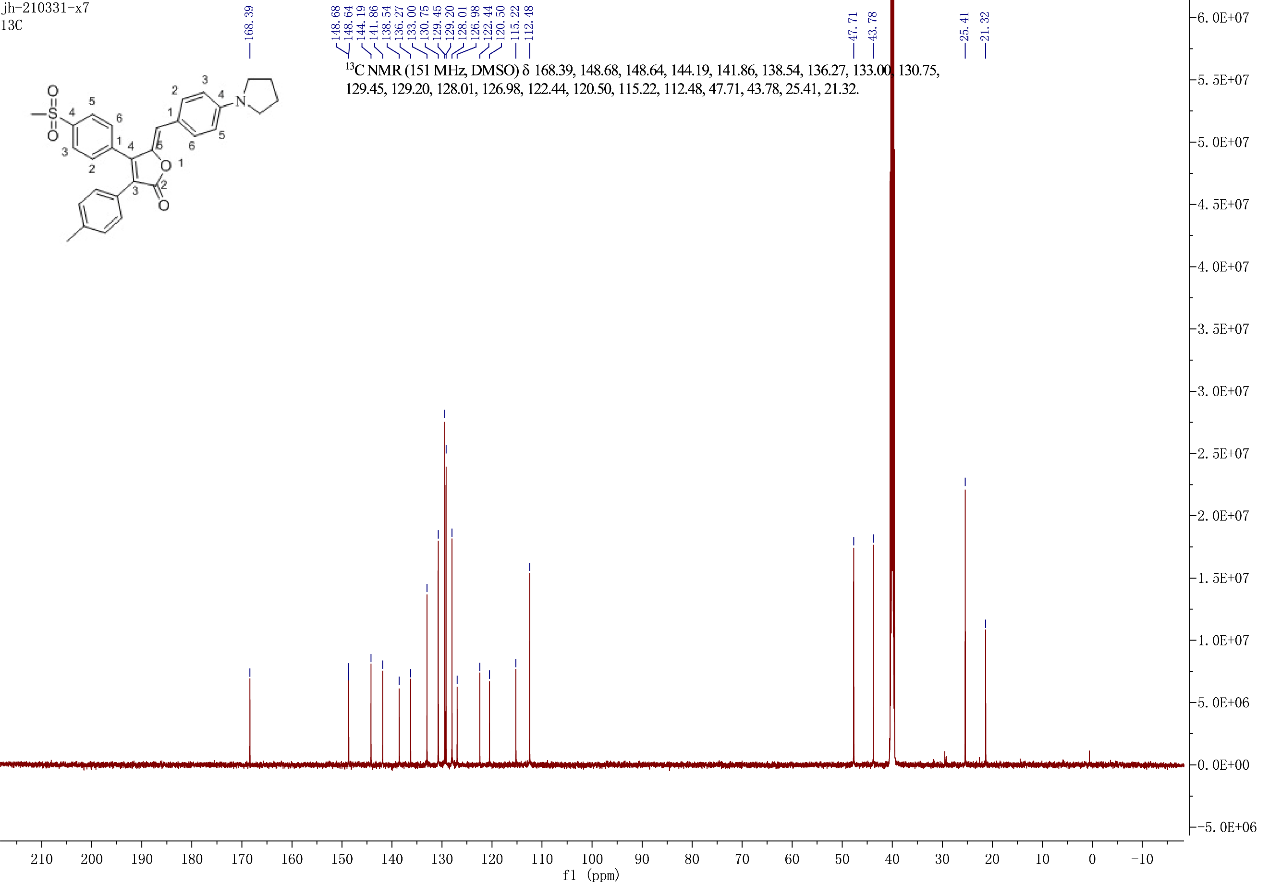


**Figure S5.** ^13^C NMR spectrum of **MOX4** in DMSO-*d_6_*.


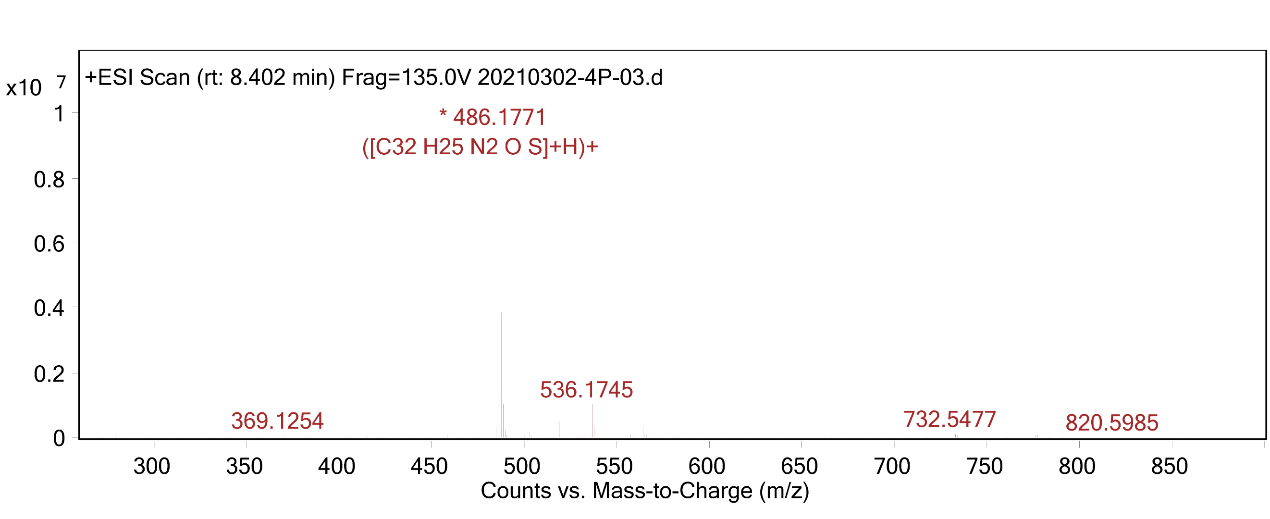


**Figure S6.** HRMS spectrum of **MOX4.**

**Beer-Lambert’s plot and linear fitting of MOX2 and MOX4**

**
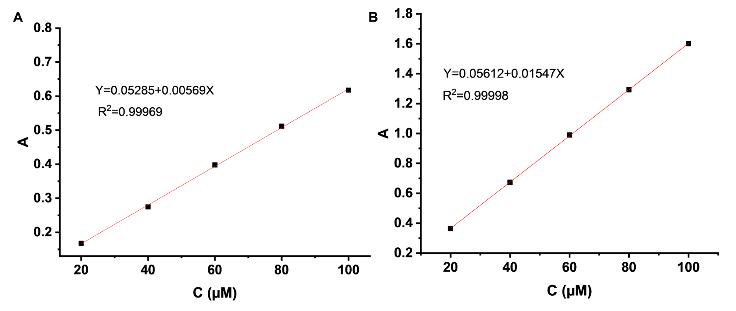
**

**Figure S7.** Beer-Lambert’s plot and linear fitting of **MOX2** (A) and **MOX4** (B). (Y=0.05285+0.00569X, R^2^=0.99969; Y=0.05612+0.01547X, R^2^=0.99998)

**The normalized absorption spectra of compounds in different solvents**


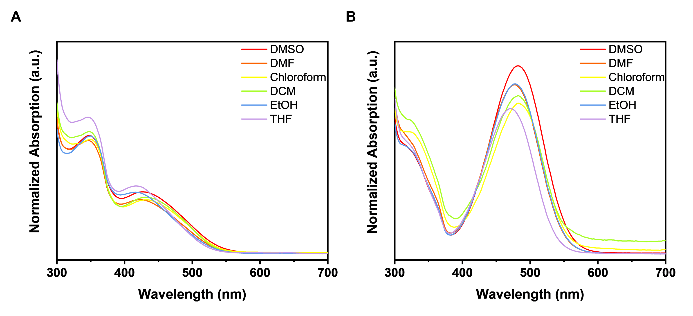


**Figure S8.** The normalized absorption spectra of **MOX2** (A) and **MOX4** (B) (5 × 10^−5^mol/L) in in different solvents.

**Table S1.** The photo-physical data of **MOX2** and **MOX4** in different solvents

|  | Solvents | *λ_abs_*(nm)^a^ | *λ_em_*(nm)^b^ | Stokes shifts(nm)^d^ | Δv(cm^-1^)^c^ |
| --- | --- | --- | --- | --- | --- |
| **MOX2** | Chloroform | 350 | 626 | 276 | 12597 |
|  | DCM | 348 | 642 | 294 | 13159 |
|  | THF | 346 | 642 | 296 | 13325 |
|  | EtOH | 350 | 626 | 276 | 12597 |
|  | DMF | 346 | 680 | 334 | 14196 |
|  | DMSO | 348 | 664 | 316 | 13675 |
| **MOX4** | Chloroform | 482 | 598 | 116 | 4024 |
|  | DCM | 482 | 622 | 140 | 4670 |
|  | THF | 472 | 624 | 152 | 5161 |
|  | EtOH | 478 | 626 | 148 | 4946 |
|  | DMF | 476 | 642 | 166 | 5432 |
|  | DMSO | 482 | 656 | 174 | 5503 |

**^a^ Absorption maxima, ^b^ Fluorescence emission maxima, ^c^ Stokes shifts, ^d^ Δv, were calculated using the equation 1/*λ_abs_*-1/*λ_em_***

**Aggregation-induced emission**


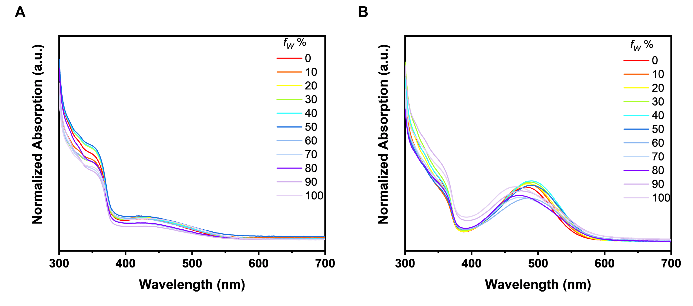


**Figure S9.** (A) The absorption spectra in function of the water fraction of **MOX2** in DMSO. (B) The absorption spectra in function of the water fraction of **MOX4** in DMSO.

**Molecular conformation of MOX2**

**
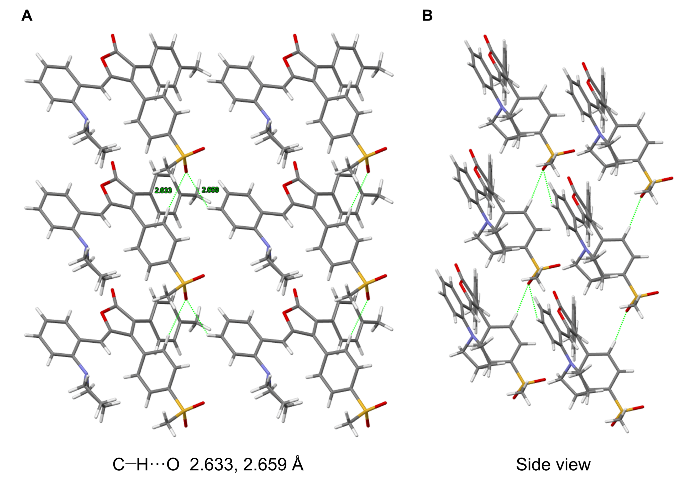
**

**Figure S10.** (A) Various intermolecular interactions in crystals of **MOX2**. (B) Side view of packing structure of **MOX2** crystal.

**Table S2.** Crystal data and structure refinement for **MOX2**

| Identification code | mo_X02_0m_a |
| --- | --- |
| Empirical formula | C_29_H_27_NO_4_S |
| Formula weight | 485.57 |
| Temperature/K | 169.98 |
| Crystal system | monoclinic |
| Space group | P2_1_/n |
| a/Å | 11.8043(11) |
| b/Å | 7.8969(7) |
| c/Å | 26.252(2) |
| α/° | 90 |
| β/° | 102.848(3) |
| γ/° | 90 |
| Volume/Å^3^ | 2385.8(4) |
| Z | 4 |
| ρ_calc_g/cm^3^ | 1.352 |
| μ/mm^‑1^ | 0.173 |
| F(000) | 1024.0 |
| Crystal size/mm^3^ | ? × ? × ? |
| Radiation | MoKα (λ = 0.71073) |
| 2Θ range for data collection/° | 5.274 to 55.004 |
| Index ranges | -12 ≤ h ≤ 15, -10 ≤ k ≤ 10, -34 ≤ l ≤ 34 |
| Reflections collected | 28763 |
| Independent reflections | 5496 [R_int_ = 0.1327, R_sigma_ = 0.0909] |
| Data/restraints/parameters | 5496/0/318 |
| Goodness-of-fit on F^2^ | 1.026 |
| Final R indexes [I>=2σ (I)] | R_1_ = 0.0599, wR_2_ = 0.1152 |
| Final R indexes [all data] | R_1_ = 0.1074, wR_2_ = 0.1380 |
| Largest diff. peak/hole / e Å^-3^ | 0.28/-0.33 |

**Normalized solid-state PL spectra of MOX2, MOX4 in the pristine state, grinding state**


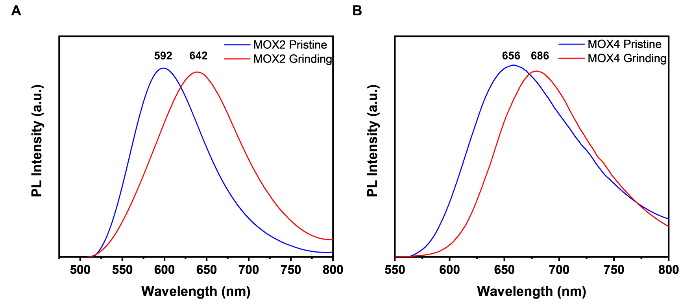


**Figure S11.** PL spectra of pristine and grinding powders of **MOX2** (A) and **MOX4** (B), excited at 365 nm.

**DSC curves of MOX2 and MOX4**


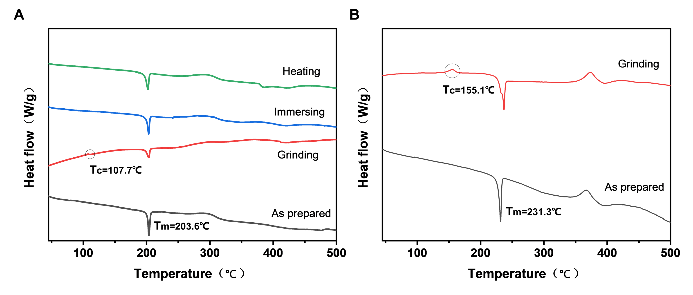


**Figure S12.** (A) DSC of **MOX2** in different states: as prepared (black line), grinding (red line), immersing with acetone (blue line) and heating (green line). (B) DSC of **MOX4** in different states: as prepared (black line), grinding (red line).

**Sensing properties toward protonic acids**


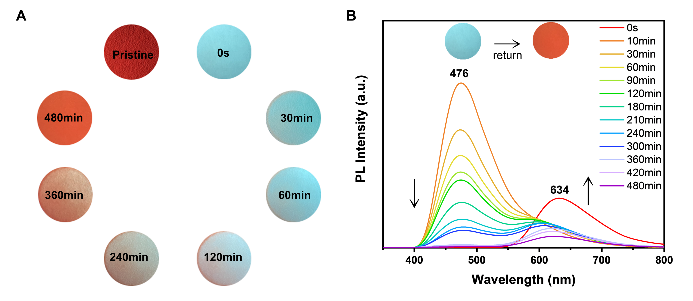


**Figure S13.** (A) The color change process of **MOX2** (500μM) in DCM solvent coated on filter paper with gradually decrease of TFA concentration. (B) PL spectra of **MOX2** (500μM) in DCM solvent coated on filter paper with gradually decrease of TFA concentration and the color change process on filter paper taken under a 365 nm hand-held UV lamp showed in the inset.

**Cell Viability Assay**


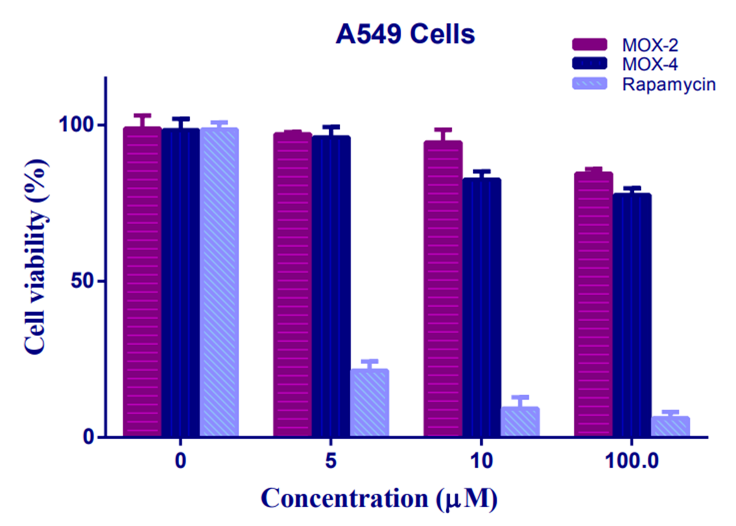


**Figure** **S14.** Cell viability values (%) estimated by MTT assays using A549 cells, cultured in the presence of 0-100.0 μM of **MOX2**, **MOX4** and Rapamycin for 24 h at 37 °C

**Lipid droplets (LDs) of MOX2**


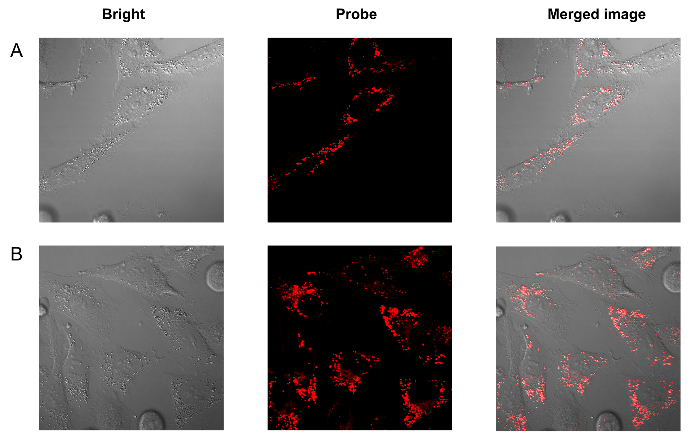


**Figure** **S15.** The confocal images of Hela cells in different emission channels with **MOX2** (A), **MOX4** (B).
